# Pan-HSV-2 IgG Antibody in Vaccinated Mice and Guinea Pigs Correlates with Protection against Herpes Simplex Virus 2

**DOI:** 10.1371/journal.pone.0065523

**Published:** 2013-06-06

**Authors:** William P. Halford, Joshua Geltz, Edward Gershburg

**Affiliations:** Department of Microbiology and Immunology, Southern Illinois University School of Medicine, Springfield, Illinois, United States of America; UC Irvine Medical Center, United States of America

## Abstract

We lack a correlate of immunity to herpes simplex virus 2 (HSV-2) that may be used to differentiate whether a HSV-2 vaccine elicits robust or anemic protection against genital herpes. This gap in knowledge is often attributed to a failure to measure the correct component of the adaptive immune response to HSV-2. However, efforts to identify a correlate of immunity have focused on subunit vaccines that contain less than 3% of HSV-2's 40,000-amino-acid proteome. We were interested to determine if a correlate of immunity might be more readily identified if ***1.*** animals were immunized with a *polyvalent* immunogen such as a live virus and/or ***2.*** the magnitude of the vaccine-induced immune response was gauged in terms of the IgG antibody response to all of HSV-2's antigens (pan-HSV-2 IgG). Pre-challenge pan-HSV-2 IgG levels and protection against HSV-2 were compared in mice and/or guinea pigs immunized with a gD-2 subunit vaccine, wild-type HSV-2, or one of several attenuated HSV-2 *ICP0* ^−^ viruses (0Δ254, 0Δ810, 0ΔRING, or 0ΔNLS). These six HSV-2 immunogens elicited a wide range of pan-HSV-2 IgG levels spanning an ∼500-fold range. For 5 of the 6 immunogens tested, pre-challenge levels of pan-HSV-2 IgG quantitatively correlated with reductions in HSV-2 challenge virus shedding and increased survival frequency following HSV-2 challenge. Collectively, the results suggest that pan-HSV-2 IgG levels may provide a simple and useful screening tool for evaluating the potential of a HSV-2 vaccine candidate to elicit protection against HSV-2 genital herpes.

## Introduction

Many herpes simplex virus 2 (HSV-2) vaccine candidates have been proposed, and most may be grouped into one of four classes: ***1.*** adjuvanted HSV-2 proteins [1]–[7]; ***2.*** HSV-2 antigen-expressing gene-delivery vectors [8]–[17]; ***3.*** inactivated HSV-2 virions [18]–[21]; or ***4.*** attenuated HSV-2 viruses [22]–[31]. The most studied HSV-2 vaccine to date is the Herpevac® vaccine, which combines HSV-2's glycoprotein D (gD-2) antigen with monophosphoryl lipid A (MPL) and alum adjuvant [3], [5]. HSV-2 glycoprotein subunit vaccines have failed to protect humans from acquiring genital herpes in several clinical trials [32]–[37]. In the most recent of these phase III clinical trials, 3,798 women immunized with an adjuvanted gD-2 vaccine acquired HSV-2 genital herpes at the same rate as 3,076 placebo-treated controls [32], [38]. These failures have raised concerns that a HSV-2 vaccine may not be tenable [38]–[40]. However, we would suggest that such speculation is premature. Several HSV-2 vaccines elicit greater protection than gD-2 vaccines in animal models [18], [23], [29], [31], but have not been evaluated in clinical trials.

Efforts to evaluate HSV-2 vaccine candidates have been hindered by the absence of a correlate of immunity that may be used to predict the quality of vaccine-induced protection [41]. The definition of the term *correlate of immunity* merits consideration, as the term has been used to convey more than one meaning. In the field of herpes immunology, the term correlate of immunity has been used to describe activities of the adaptive immune response that temporally correlate with the cessation of HSV-2 replication. For example, genital herpes lesions in human subjects cease to produce infectious HSV-2 at a time that precisely correlates with the infiltration of CD8^+^ T-cells and detection of IFN-γ in these lesions [42]. These and other observations provide strong evidence that T-cells are a critical effector of host control of HSV-2 infections *in vivo* (reviewed in Ref. [43]–[45]).

The second meaning of the term *correlate of immunity* relates to its broader use in the vaccine literature [46]. A correlate of immunity generally refers to a parameter whose magnitude correlates with the quality of protective immunity regardless of its role in mediating protection. For example, antigen-specific antibodies are a useful correlate of immunity for the vaccines used to prevent yellow fever, measles, and chickenpox [46]. T-cells are likely critical to vaccine-induced protection against these viral diseases. Nonetheless, high antibody titers provide a reliable basis for predicting which vaccine recipients are well protected against yellow fever, measles, or chickenpox versus individuals with low antibody titers who remain susceptible and may require re-vaccination [46]. In the current study, the term *correlate of immunity* is intended to convey this latter meaning.

A correlate of immunity would be valuable if it provided a basis to differentiate which HSV-2 vaccine candidates [1]–[31] are most effective at eliciting protection against HSV-2. In animal models, two standard metrics of protection against HSV-2 are reductions in HSV-2 challenge virus shedding, and increased survival frequency following a lethal HSV-2 challenge [1], [16], [23], [29], [47]. The recent review of Dropulic and Cohen [41] succinctly summarizes what is currently known about correlates of immunity to HSV-2: *“The correlates of protection for a prophylactic HSV-2 vaccine are unknown at present”.*

A recent study by a major pharmaceutical company brings the problem into focus. This study was performed to test a hypothesis that a gD-2 subunit vaccine would be more protective if it elicited a more potent T-cell response against HSV-2 glycoprotein antigens [6], [48]. To test this hypothesis, the T-cell response was increased by replacing alum/MPL adjuvant with alum/CpG oligonucleotide adjuvant, and by adding a dominant HSV-2 T-cell antigen, glycoprotein B-2 (gB-2), to the vaccine [49]–[51]. These modifications yielded a >10-fold increase in the frequency of HSV-2-specific T-cells, but the efficacy of vaccine-induced protection against HSV-2 did not improve [6]. Consequently, the Pfizer Research Group concluded that “*difficulties in correlating immune responses to efficacy in animal models will act as a deterrent to researchers attempting to develop effective HSV vaccines*” [6].

It has been suggested that past difficulties in identifying a clinically useful correlate of immunity to HSV-2 may have stemmed from a failure to identify the correct parameter of the T-cell response that controls HSV-2 *in vivo* [48]. However, there is a second possibility. Most attempts to identify a correlate of immunity to HSV-2 have focused on monovalent (gD-2) or bivalent (gB-2+ gD-2) subunit vaccines that present less than 3% of HSV-2′s 40,000 amino-acid proteome to the immune system [1]–[17]. This approach does not consider HSV-2's full complement of antigens; at least 20 viral proteins are known targets of the human B- and T-cell response to HSV-2 [49], [51], [52]. Therefore, we postulated that a correlate of immunity might be more readily identified if ***1.*** animals were immunized with a *polyvalent* immunogen such as a live virus and/or ***2.*** the magnitude of the vaccine-induced immune response was gauged in terms of the IgG antibody response to all of HSV-2's antigens (pan-HSV-2 IgG).

The current study was initiated to test these predictions. A novel, flow cytometry-based assay was developed to measure pan-HSV-2 IgG levels. Using this assay, 117 naïve and immunized animals were analyzed to compare pre-challenge serum levels of pan-HSV-2 IgG to two measures of protection against HSV-2. In both mice and guinea pigs, we observed that increased pre-challenge levels of pan-HSV-2 IgG quantitatively correlated with ***1.*** reductions in HSV-2 challenge virus shedding and ***2.*** increased survival frequency following a lethal HSV-2 challenge. These results suggest that serum levels of pan-HSV-2 IgG antibody may provide a simple and useful screening tool to aid in the identification of a HSV-2 vaccine that elicits robust protection against HSV-2 genital herpes.

## Results

### A Flow Cytometry-based Assay to Measure Pan-HSV-2 IgG Antibody Levels

**Figure 1:**
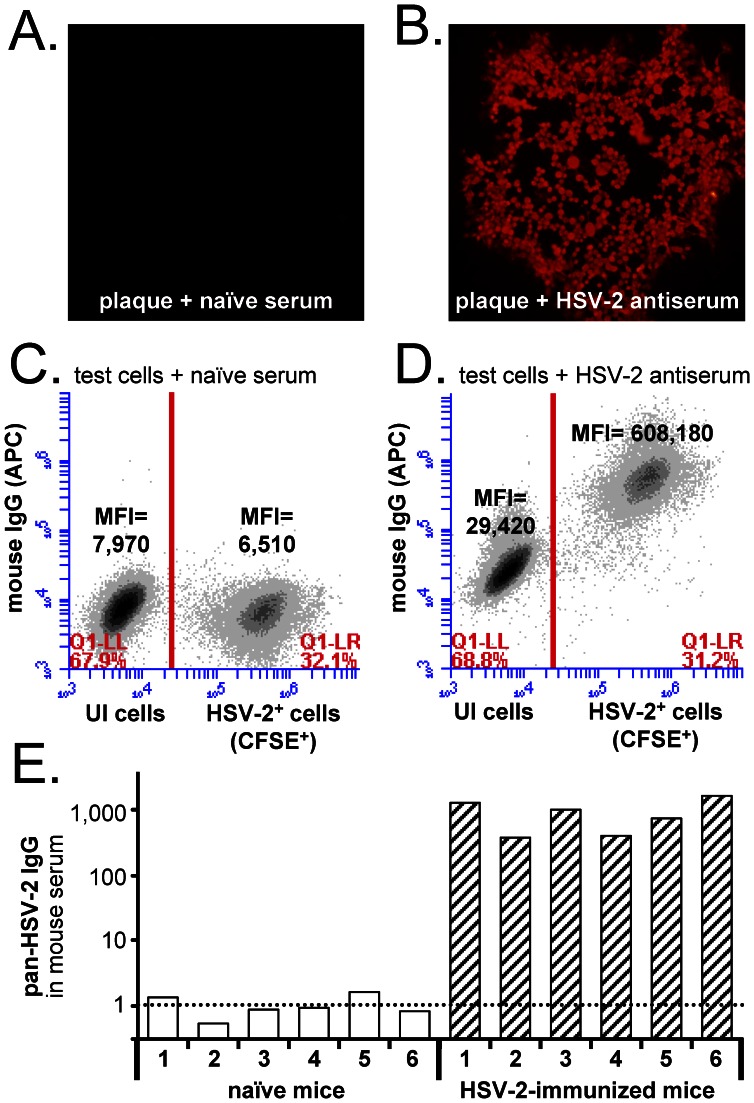
Flow cytometry-based measurement of pan-HSV-2 IgG antibody levels. **A and B.** Immunofluorescent labeling of fixed HSV-2 plaques with a 1∶6,000 dilution of (**A**) naïve mouse serum or (**B**) HSV-2 antiserum obtained from mice immunized with HSV-2 0ΔNLS [53]. Mouse IgG binding was visualized with AlexaFluor594-labeled goat anti-mouse IgG (H+L). **C and D.** Two-color flow cytometric analysis of a fixed, single-cell suspension of CFSE-labeled, HSV-2-infected (HSV-2^+^) Vero cells mixed with uninfected (UI) Vero cells. Fixed cells were incubated with a 1∶6,000 dilution of (**C**) naïve mouse serum or (**D**) mouse HSV-2 antiserum and APC-labeled goat anti-mouse IgG, and were analyzed for CFSE (FL1) and APC (FL4) fluorescent intensity. **E.** Pan-HSV-2 IgG levels in the serum of n = 6 naïve mice versus n = 6 HSV-2 0ΔNLS-immunized mice, as determined by the ΔMFI between HSV-2^+^ and UI cells.

The presence of serum IgG antibodies that bind total HSV-2 antigens (pan-HSV-2 IgG) may be qualitatively tested by immunofluorescent staining of HSV-2 plaques in fixed Vero cell monolayers (Fig. 1A, B). A more quantitative, flow-cytometry-based variant of this assay was developed. Single-cell suspensions of HSV-2-infected (HSV-2^+^) and uninfected (UI) Vero cells were obtained by dispersing culture monolayers, fixing and permeabilizing cells, and filtering through 40 µm mesh and a 25-g needle to remove cell clumps. To permit antibody staining of HSV-2^+^ versus UI cells in a single reaction, HSV-2^+^ cells were labeled with the green fluorophore carboxyfluorescein diacetate, succinimidyl ester (CFSE).

Suspensions of ∼30% HSV-2^+^ cells and ∼70% UI cells were incubated with serum from naïve mice or HSV-2-immunized mice, and were fluorescently labeled with allophycocyanin (APC)-anti-mouse IgG Fc fragment secondary antibody. Antibody-labeled cells were analyzed by 2-color flow cytometry (Fig. 1C, D). When cell suspensions were incubated with a 1∶6,000 dilution of naïve mouse serum, similar levels of IgG antibody bound HSV-2^+^ cells and UI cells (HSV_MFI_ = 6,510; UI_MFI_ = 7,970; Fig. 1C). In contrast, when cell suspensions were incubated with a 1∶6,000 dilution of HSV-2 antiserum, the level of antibody bound to HSV-2^+^ cells was much higher than UI cells (HSV_MFI_ = 608,180; UI_MFI_ = 29,420; Fig. 1D).

Mouse serum levels of “pan-HSV-2 IgG” antibody were estimated based on the difference in mean fluorescence intensity (ΔMFI) between HSV-2^+^ cells versus UI cells. The resulting ΔMFI-value associated with each serum sample was normalized to a “fold-increase above background” by the following calcluation: *ΔMFI_ test sample_ ÷ average ΔMFI _naïve sera_*. When this approach was applied, sera from n = 6 naïve mice were estimated to possess pan-HSV-2 IgG levels that were 1.0±0.2 times background (Fig. 1E). In contrast, n = 6 mice immunized with a live-attenuated HSV-2 0ΔNLS virus [29] possessed levels of pan-HSV-2 IgG that were 940±240 times background (Fig. 1E). Therefore, flow cytometry of antibody-stained HSV-2^+^ versus UI cells provided a potential means to measure pan-HSV-2 IgG abundance in the serum of vaccinated animals.

### Comparison of Methods for Enumerating Serum Levels of HSV-2-specific Antibody

Flow cytometry-based measurements of pan-HSV-2 IgG abundance were compared to two more traditional assays; namely, a HSV-2 neutralization assay and an antibody-capture ELISA. For this comparison, an antiserum dilution series was constructed by diluting mouse HSV-2 antiserum into naïve serum in 0.33-log increments spanning a 4,640-fold range. The use of naïve mouse serum as a diluent ensured that serum protein concentration (e.g., IgG) remained constant while HSV-2 specific antibodies were selectively diluted out in 0.33-log increments.

HSV-2 antiserum neutralized the infectivity of HSV-2 between dilutions of 1∶21 and 1∶1,000, and exhibited little to no neutralizing activity at 1∶2,150 or greater dilutions (Fig. S1A). Thus, the dynamic range of the HSV-2 neutralization assay was 1∶21 to 1∶1,000, and the coefficient of variation of measurements was 16±8% within this range (Table 1).

**Table 1:** Comparison of three methods used to measure serum levels of HSV-2-specific antibodies.

	Neutralization assay	ELISA (total HSV-2 Ag)	flow cytometry assay
Linear range a	1∶21–1∶1,000	1∶100–1∶100,000	1∶6,000–1∶6,000,000
Coefficient of variation b	16±8%	13±3%	5±1%
Goodness-of-fit c (linear regression)	r^2^ = 1.00 (p<10^−5^)	r^2^ = 1.00 (p<10^−10^)	r^2^ = 1.00 (p<10^−12^)

a Range of HSV-2 antiserum dilutions in which estimates of anti-HSV-2 antibody abundance changed in linear relation to changes in serum dilution.

b Mean ± sem coefficient of variation of triplicate measurements for each serum dilution in the linear range of each assay. For each serum dilution considered, the coefficient of variation = 100× standard deviation ÷ mean.

c Goodness-of-fit (r^2^) of observed data relative to values predicted by a regression model within the linear range. The p-value refers to the probability that the quantity measured by each assay (i.e., neutralizing titer, OD_405_, or ΔMFI) did not vary as a function of HSV-2 antiserum dilution.

HSV-2 antibody abundance in the antiserum dilution series was evaluated by antibody-capture ELISA using lysates of HSV-2-infected Vero cells as a coating antigen. Antibody capture-ELISA yielded significant conversion of para-nitrophenylphosphate substrate (OD_405_) at serum dilutions between 1∶100 and 1∶100,000 (Fig. S1B). In this linear range, the coefficient of variation of ELISA-based measurement of pan-HSV-2 IgG levels was 13±3% (Table 1).

HSV-2 antibody abundance in the antiserum dilution series was evaluated by a novel, flow cytometry-based assay (Fig. 1). Flow cytometry of serum-stained test cells yielded a significant ΔMFI of IgG antibody-binding to HSV-2^+^ cells versus UI cells between 1∶6,000 and 1∶6,000,000 dilutions of antiserum (Fig. S1C). In this linear range, the coefficient of variation of flow cytometry-based measurements of pan-HSV-2 IgG leverls was 5±1% (Table 1).

All three assays yielded parallel estimates of pan-HSV-2 antibody abundance, but the flow cytometry-based assay was the most sensitive. Specifically, the flow assay had a lower limit-of-detection of 1∶6,000,000 relative to HSV-2 antiserum, whereas the HSV-2 neutralization assay and antibody-capture ELISA had lower limits of 1∶2,100 and 1∶100,000, respectively (Table 1). In addition, the flow cytometry-based assay was the most precise, and exhibited a 2- to 3-fold lower coefficient of variation relative to the other assays (Table 1). Finally, the flow cytometry-based assay was unique in that the primary metric, ΔMFI, represented the average IgG antibody binding to 25,000 HSV-2^+^ cells versus ∼50,000 background control cells. This extensive replication in measurements accounted for the increased precision of the flow cytometry-based method.

### Pan-HSV-2 IgG Correlates with Protection Against Ocular HSV-2 Challenge in Mice

A retrospective analysis was performed on n = 48 serum samples derived from mice used in a previously published ocular HSV-2 challenge experiment (Figs. 5 and 6 in Ref. [53]). The goal of this analysis was to determine if pan-HSV-2 IgG levels in archived sera varied in proportion to the protection observed in mice ocularly challenged with HSV-2.

**Figure 2:**
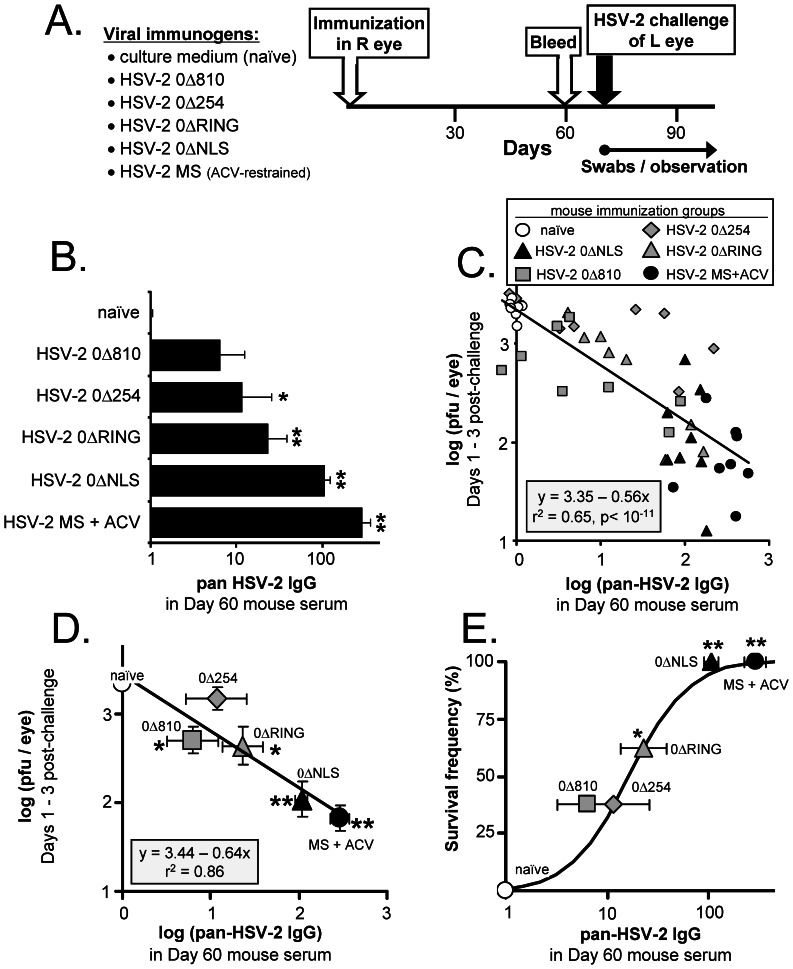
Pan-HSV-2 IgG levels correlate with protection against ocular HSV-2 challenge in mice. (**A**) Design of vaccine-ocular HSV-2 challenge experiment in mice. Mice were initially inoculated in their right eye on Day 0 with culture medium or 10^5^ pfu per eye of one of the five indicated viruses (n = 8 per group). Mice inoculated with HSV-2 MS were treated with acyclovir from Days 0 to 20 post-immunization to restrict viral pathogenesis. On Day 60, blood was harvested, and on Day 70, mice were challenged in the left eye with 10^5^ pfu of wild-type HSV-2 MS. (**B**) Mean ± sem pan-HSV-2 IgG levels in pre-challenge serum, as determined by a flow cytometry-based assay. (**C**) For each mouse (one symbol per mouse), the average amount of infectious HSV-2 shed on Days 1, 2, and 3-post ocular challenge (y-axis) was plotted as a function of the pre-challenge HSV-2 IgG levels observed in the same mouse (x-axis). The solid black line represents the best-fit linear regression model, y = 3.35–0.56x, for the 48 matched datum pairs. (D) Mean ± sem of log (pan-HSV-2 IgG) in each immunization group is plotted on the x-axis versus mean ± sem ocular HSV-2 shedding on the y-axis. The solid black line represents the best-fit linear regression model, y = 3.44–0.64x, for these 6 matched averages (r^2^ = 0.86). Groups of immunized mice that exhibited a significant reduction in ocular HSV-2 shedding relative to naïve mice are indicated by a single asterisk (*; p<0.05) or double-asterisk (**; p<0.001), as determined by one-way ANOVA and Tukey's post-hoc t-test. (**E**) Survival frequency in each group is plotted as a function of the mean ± sem pan-HSV-2 IgG antibody level observed in each group. Groups of immunized mice that exhibited a significant difference in survival frequency relative to naïve mice are indicated by a single asterisk (*; p<0.05) or double-asterisk (**; p<0.0001), as determined by Fisher's Exact Test.

The design of the original experiment is reviewed. Five of 6 groups of mice were inoculated in the *right* eye with culture medium (naïve controls) or 100,000 pfu per *right* eye of the HSV-2 *ICP0* ^−^ mutant viruses HSV-2 0ΔNLS, 0Δ810, 0Δ254, or 0ΔRING (Fig. 2A). A sixth group was similarly inoculated with wild-type HSV-2 MS, but the pathogenesis of infection was restrained by treating mice with acyclovir (Fig. 2A). Blood was drawn on Day 60, and mice were challenged on Day 70 with 100,000 pfu per *left eye* of HSV-2 MS (Fig. 2A). The left eyes of these mice were swabbed daily between Days 1 and 3 post-challenge to monitor viral replication, and disease onset was observed over a 30 day-period (Fig. 2A).

Pre-challenge levels of pan-HSV-2 IgG in the immunization groups were determined and rank-ordered (Fig. 2B). Mice immunized with the HSV-2 0Δ810, 0Δ254, or 0ΔRING viruses possessed low to intermediate levels of pan-HSV-2 IgG that were an average 5- to 23-fold above background (Fig. 2B). In contrast, mice immunized with HSV-2 0ΔNLS or acyclovir-restrained HSV-2 MS possessed pan-HSV-2 IgG levels that were an average 110- and 290-fold above background, respectively (Fig. 2B).

Regression analysis was applied to determine if pre-challenge pan-HSV-2 IgG levels correlated with reduced HSV-2 shedding after ocular challenge. The null hypothesis predicted that the best-fit linear regression model (y = b+mx) for these 48 matched datum pairs would have a slope (m) of 0 (Fig. 2C). The probability that this null hypothesis was correct was p<10^−11^. Rather, HSV-2 challenge virus shedding (y-variable) decreased an average 0.56 logarithms for every l logarithm that pan-HSV-2 IgG levels (x-variable) increased (black line in Fig. 2C).

The goodness-of-fit (r^2^) value for the best-fit linear regression model was 0.65, which reflected the fact that the observed level of HSV-2 shedding in many mice did not conform perfectly to the quantity predicted by the equation y = 3.35–0.56x (black line in Fig. 2C). However, the average level of ocular HSV-2 shedding decreased in direct proportion to pan-HSV-2 IgG levels in 5 of 6 immunization groups, within the standard error of the measurements (Fig. 2D; r^2^ = 0.86). The exception to this trend was mice immunized with the HSV-2 0Δ254 virus, which elicited highly variable protection against HSV-2, and was thus rapidly eliminated from consideration as a viable live HSV-2 vaccine candidate [53].

The frequency with which immunized mice survived ocular HSV-2 challenge was plotted as a function of pre-challenge pan-HSV-2 IgG levels (Fig. 2E). Naïve mice had undetectable levels of pan-HSV-2 IgG, and none survived HSV-2 challenge (Fig. 2E). Mice immunized with HSV-2 0Δ810 or HSV-2 0Δ254 had the lowest levels of pan-HSV-2 IgG, and only 3 of 8 (43%) per group survived HSV-2 challenge (Fig. 2E). Mice immunized with HSV-2 0ΔRING had intermediate pan-HSV-2 IgG levels, and 5 of 8 survived HSV-2 challenge (Fig. 2E). Mice immunized with HSV-2 0ΔNLS or acyclovir-restrained MS had the highest pre-challenge levels of pan-HSV-2 IgG, and 100% survived ocular HSV-2 challenge (Fig. 2E). Collectively, these results indicated that pre-challenge pan-HSV-2 IgG levels correlated with vaccine-induced protection against HSV-2 in terms of ***1.*** reduced ocular shedding of the HSV-2 challenge virus and ***2.*** increased survival frequency.

### ELISA- Versus Flow Cytometry-estimates of Pan-HSV-2 IgG Levels

A test was conducted to determine if flow cytometry measurement of pan-HSV-2 IgG levels offered any practical advantage relative to antibody-capture ELISA. To this end, the same mouse serum samples considered above were re-analyzed by antibody-capture ELISA using HSV-2-infected cell lysates as coating antigen. A 0.33-log dilution series of HSV-2 antiserum was used to precisely define the sigmoidal relationship between OD_405_ absorbance values and log (pan-HSV-2 IgG) levels using a hyperbolic tangent equation (Fig. S2A; r^2^ = 1.00). Estimates of log (pan-HSV-2 IgG) levels for each serum sample were mathematically derived by fitting each serum sample’s OD_405_ absorbance values to this standard curve.

ELISA-based estimates of log (pan-HSV-2 IgG) correlated with decreased ocular HSV-2 shedding (black line in Fig. S2B; r^2^ = 0.54). However, the goodness-of-fit of ELISA estimates of pan-HSV-2 IgG was less robust than the equivalent correlation with flow cytometry estimates of pan-HSV-2 IgG (Fig. 2C; r^2^ = 0.65). In part, this was due to the 2.5-fold higher variance of ELISA- versus flow cytometry-based estimates of pan-HSV-2 IgG (Table 1).

The relative sensitivity of ELISA versus flow cytometry estimates of pan-HSV-2 IgG was graphically analyzed. ELISA estimates of log (pan-HSV-2 IgG) were plotted on the x-axis, whereas the corresponding flow cytometry estimates were plotted on the y-axis (Fig. S2C). If ELISA- versus flow cytometry-estimates were equally sensitive, then these n = 48 datum points should graphically scatter around a '0 log' line-of-equivalence (Fig. S2C). However, 35 of 36 seropositive samples fell above the line-of-equivalence suggesting that flow cytometry yielded higher estimates of log (pan-HSV-2 IgG) than ELISA. In 6 of the 36 seropositive samples, flow cytometry yielded a more than +1 log-higher estimate of pan-HSV-2 IgG relative to ELISA (Fig. S2C). In these n = 36 seropositive samples, flow cytometry yielded an average 5±1-fold higher estimate of pan-HSV-2 IgG level relative to ELISA. At the two extremes of pan-HSV-2 IgG levels, datum points clustered near the line of equivalence (Fig. S2C). However, in the low- to mid-range of sensitivity, the flow-cytometry assay was more sensitive than ELISA (p<0.01 for HSV-2 0ΔNLS, 0Δ810, 0Δ254, or 0ΔRING; paired t-test). Based on this and earlier analyses (Table 1), we concluded that flow cytometry and ELISA yielded parallel estimates of pan-HSV-2 IgG levels, but the flow cytometry method offered improved precision and sensitivity.

### Pan-HSV-2 IgG Correlates with Protection against Vaginal HSV-2 Challenge in Mice

A second, retrospective analysis was performed on mouse serum derived from a previously published experiment (Fig. 4 in Ref. [29]). The goal of this analysis was to determine if pan-HSV-2 IgG levels in archived sera varied in proportion to the protection observed in mice vaginally challenged with HSV-2.

**Figure 3:**
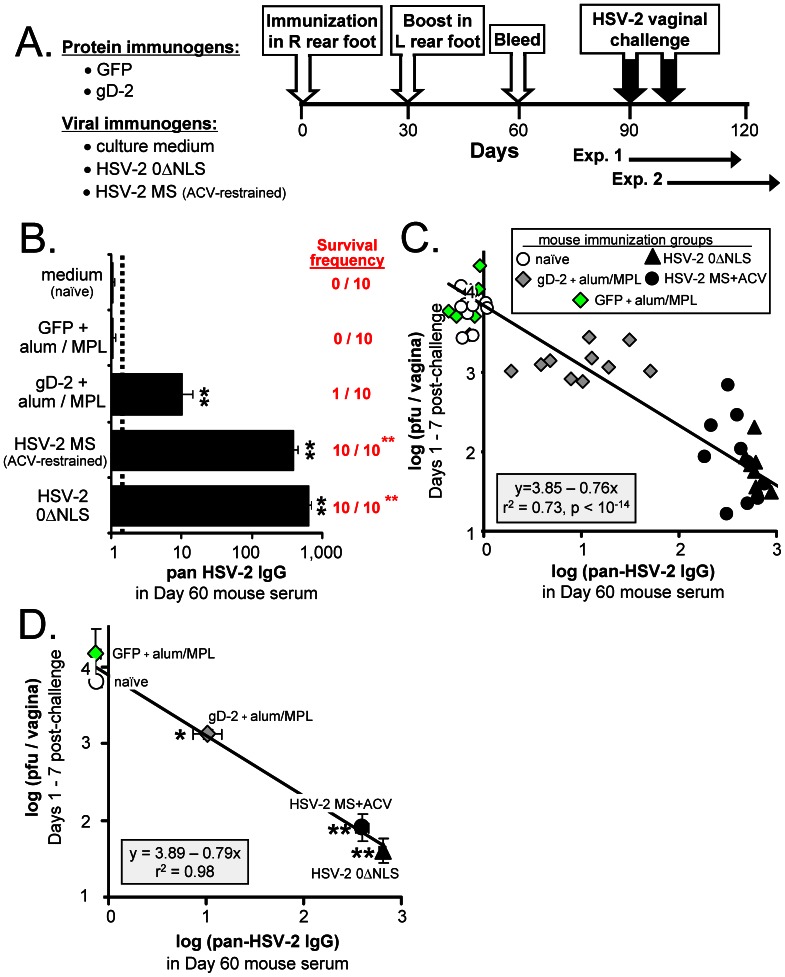
Pan-HSV-2 IgG levels correlate with protection against vaginal HSV-2 challenge in mice. (**A**) Design of mouse vaccine-challenge experiment. Mice were immunized in their right, rear footpads on Day 0 with gD-2, GFP, culture medium (mock), HSV-2 0ΔNLS, or HSV-2 MS, as described in the Results (n = 10 per group). Mice immunized with HSV-2 MS received 1 mg/ml acyclovir in drinking water from Days 0 to 20 post-immunization to restrain the pathogenesis of a primary exposure to wild-type HSV-2. All mice were boosted in their left, rear footpads on Day 30 with an equivalent, booster immunization with the exception that MS-immunized mice did not require acyclovir during the boost. On Day 60, blood was harvested, and on Days 90 or 100, mice were challenged with 500,000 pfu per vagina of wild-type HSV-2 MS. Seven and 3 days prior to HSV-2 MS challenge, each mouse received a subcutaneous injection of 2 mg DepoProvera® (medoxyprogesterone) to render mouse vaginas susceptible to HSV-2 challenge. (**B**) Mean ± sem pan-HSV-2 IgG levels in pre-challenge serum, as determined by a flow cytometry-based assay. The frequency with which mice survived until Day 30 post-challenge is indicated. (**C**) For each mouse (one symbol per animal), the average amount of infectious HSV-2 shed on Days 1, 3, 5, and 7 post-vaginal challenge (y-axis) was plotted as a function of pre-challenge pan-HSV-2 IgG levels observed in the same mouse (x-axis). The solid black line represents the best-fit linear regression model, y = 3.85–0.76x, for the 50 matched datum pairs. (**D**) Mean ± sem of log (pan-HSV-2 IgG) in each immunization group is plotted on the x-axis versus mean ± sem vaginal HSV-2 shedding on the y-axis. The solid black line represents the best-fit linear regression model, y = 3.89–0.79x, for these 5 matched averages (r^2^ = 0.98). Groups of immunized mice that exhibited a significant reduction in vaginal HSV-2 shedding relative to naïve mice are indicated by a single asterisk (*; p<0.05) or double-asterisk (**; p<0.001), as determined by one-way ANOVA and Tukey's post-hoc t-test.

The design of the original experiment is reviewed. Mice were immunized on Days 0 and 30 in their right and left rear footpads, respectively, with ***1.*** culture medium (naïve controls); ***2.*** 2.5 µg green fluorescent protein (GFP) adjuvanted with alum and 10 µg MPL; ***3.*** 2.5 µg gD-2_306t_ [54] adjuvanted with alum and 10 µg MPL; ***4.*** 10^6^ pfu HSV-2 0ΔNLS, or ***5.*** 10^6^ pfu wild-type HSV-2 MS where acyclovir was used to limit the pathogenesis of the primary exposure to MS (Fig. 3A; n = 10 per group). Blood was drawn on Day 60 and mice were challenged on Days 90 or 100 with 500,000 pfu per vagina of HSV-2 MS. All n = 50 mice were swabbed between Days 1 and 7 post-challenge to measure vaginal HSV-2 shedding and disease onset was observed over a 30 day-period (Fig. 3A).

Pan-HSV-2 IgG levels in the immunization groups were determined and rank-ordered (Fig. 3B). Naïve and GFP-immunized mice did not possess detectable pan-HSV-2 IgG, and none of these mice survived vaginal HSV-2 challenge (Fig. 3B). Mice immunized with gD-2 possessed pan-HSV-2 IgG that was an average 10-fold above background, and 1 of 10 survived vaginal HSV-2 challenge (Fig. 3B). Importantly, anti-gD-2-titers in gD-2-immunized mice were ∼200,000 (Fig. 3C of Ref. [29]), which is comparable to other published studies [2], [3], [5]. Mice immunized with the live HSV-2 viruses MS or 0ΔNLS possessed pre-challenge pan-HSV-2 IgG levels that were an average 390- and 650-fold above background, respectively; 100% of these mice survived vaginal HSV-2 challenge without visible symptoms of disease (Fig. 3B).

Regression analysis was applied to determine if pre-challenge pan-HSV-2 IgG levels correlated with reduced HSV-2 shedding after vaginal challenge. The null hypothesis predicted that the best-fit linear regression model for these 50 matched datum pairs would have a slope (m) of 0 (Fig. 3C). The probability that this null hypothesis was correct was p<10^−14^. Rather, HSV-2 challenge virus shedding (y) decreased an average 0.76 logarithms for every l logarithm that pan-HSV-2 IgG levels (x) increased (black line in Fig. 3C). The goodness-of-fit (r^2^) for this regression line was 0.73, which reflected the fact that the observed level of HSV-2 shedding in many mice did not conform perfectly to the quantity predicted by the equation y = 3.85–0.76x (black line in Fig. 3C). However, the average level of vaginal HSV-2 shedding decreased in direct proportion to pan-HSV-2 IgG levels in all 5 immunization groups within the standard error of the measurements (Fig. 3D; r^2^ = 0.98). Therefore, pre-challenge pan-HSV-2 IgG levels correlated with vaccine-induced protection against HSV-2 in mice in terms of ***1.*** reduced vaginal shedding of the HSV-2 challenge virus and ***2.*** increased survival frequency.

### Pan-HSV-2 IgG Correlates with Protection against Vaginal HSV-2 Challenge in Guinea Pigs

**Figure 4:**
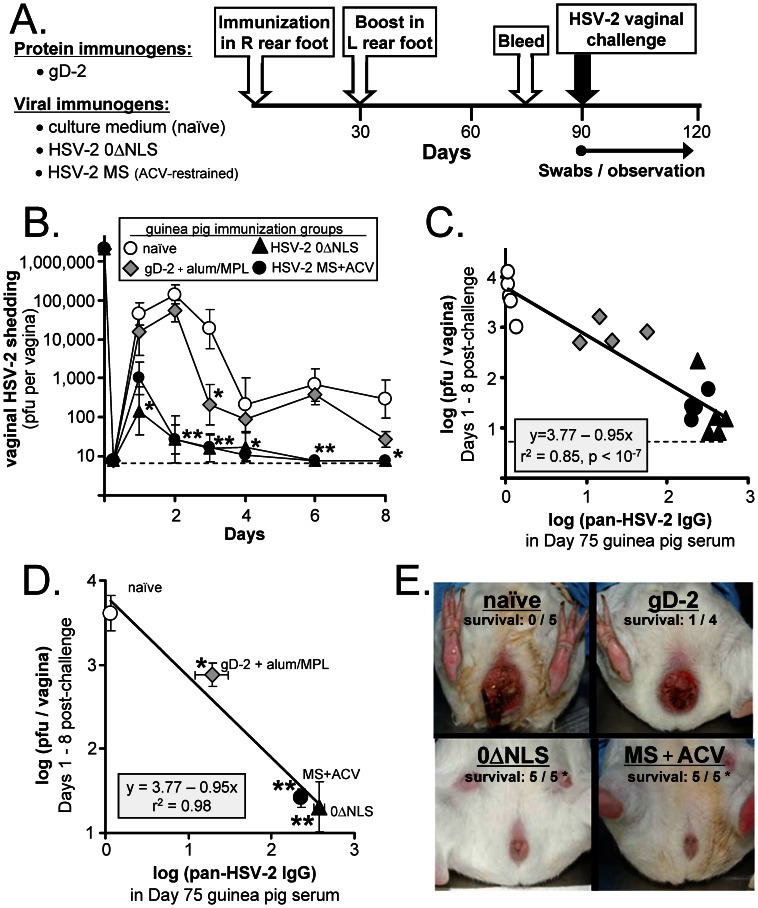
Pan-HSV-2 IgG levels correlate with protection against vaginal HSV-2 challenge in guinea pigs. (**A**) Design of guinea pig vaccine-challenge experiment. Guinea pigs were immunized in their right, rear footpads on Day 0 with gD-2, culture medium (mock), HSV-2 0ΔNLS, or HSV-2 MS, as described in the Results (n = 5 per group). Guinea pigs immunized with HSV-2 MS received 1 mg/ml acyclovir in drinking water from Days 0 to 20 post-immunization to restrain the pathogenesis of a primary exposure to wild-type HSV-2. All guinea pigs were boosted in their left, rear footpads on Day 30 with an equivalent, booster immunization; MS-immunized guinea pigs did not receive acyclovir during the secondary boost. On Day 75, blood was harvested, and on Day 90, guinea pigs were challenged with 2×10^6^ pfu per vagina of wild-type HSV-2 MS. (**B**) Mean ± sem pfu of HSV-2 shed per vagina between Days 1 and 8 post-challenge in guinea pigs that were naïve (n = 5) or were immunized with gD-2+ alum/MPL (n = 4), HSV-2 0ΔNLS (n = 5), or an acyclovir (ACV)-restrained HSV-2 MS infection (n = 5). A single asterisk (*) denotes p<0.05 and a double asterisk (**) denotes p<0.0001 that HSV-2 MS vaginal shedding was equivalent to naïve guinea pigs on that day, as determined by one-way ANOVA and Tukey’s post hoc t-test. (**C**) For each guinea pig (one symbol per animal), the average amount of infectious HSV-2 shed on Days 1, 2, 3, 4, 6, and 8 post-vaginal challenge (y-axis) was plotted as a function of pre-challenge pan-HSV-2 IgG levels observed in the same guinea pig (x-axis). The solid black line represents the best-fit linear regression model, y = 3.77–0.95x, for these 19 matched datum pairs. (**D**) Mean ± sem of log (pan-HSV-2 IgG) in each immunization group is plotted on the x-axis versus mean ± sem vaginal HSV-2 shedding on the y-axis. The solid black line represents the best-fit linear regression model, y = 3.77–0.95x, for these 4 matched averages (r^2^ = 0.98). Groups of immunized guinea pigs that exhibited a significant reduction in vaginal HSV-2 shedding relative to naïve guinea pigs are indicated by a single asterisk (*; p<0.05) or double-asterisk (**; p<0.001), as determined by one-way ANOVA and Tukey's post-hoc t-test. (**E**) The worst case of perivaginal disease in each group of naïve or immunized guinea pigs on Day 7 post-challenge. Survival frequency refers to the frequency with which animals in each immunization group survived until Day 30 post-challenge.

A third, prospective analysis was performed to determine if pre-challenge pan-HSV-2 IgG levels varied in proportion to protection against HSV-2 in a species other than mice. To address this question, groups of n = 5 guinea pigs were immunized on Days 0 and 30 in their right and left rear footpads, respectively, with ***1.*** culture medium (naïve), ***2.*** 5 µg gD-2 adjuvanted with alum and 20 µg MPL, ***3.*** 2×10^6^ pfu HSV-2 0ΔNLS, or ***4.*** 2×10^6^ pfu of wild-type HSV-2 MS where acyclovir was used to restrict the pathogenesis of the primary exposure to MS (Fig. 4A). Guinea pigs were bled on Day 75 and challenged on Day 90 with 2×10^6^ pfu HSV-2 MS per vagina (Fig. 4A). Unfortunately, one gD-2-immunized guinea pig was lost to an anesthetic overdose; thus, only n = 4 gD-2-immunized guinea pigs were available following HSV-2 vaginal challenge. Naïve guinea pigs shed peak titers of ∼200,000 pfu per vagina on Day 2 post-challenge (Fig. 4B). Guinea pigs immunized with gD-2 shed an average 5-fold less HSV-2 relative to naïve guinea pigs between Days 1 and 8 post-challenge (Fig. 4B). In contrast, guinea pigs immunized with HSV-2 MS or 0ΔNLS shed an average 150- and 200-fold less HSV-2, respectively, relative to naïve guinea pigs (Fig. 4B).

Regression analysis was applied to determine if pre-challenge pan-HSV-2 IgG levels in guinea pigs correlated with reduced HSV-2 shedding after vaginal challenge. The null hypothesis predicted that the best-fit linear regression model for these n = 19 matched datum pairs would have a slope (m) of 0 (Fig. 4C). The probability that this null hypothesis was correct was p<10^−7^. Rather, HSV-2 challenge virus shedding (y) decreased an average 0.95 logarithms for every l logarithm that pan-HSV-2 IgG levels (x) increased (black line in Fig. 4C). The goodness-of-fit (r^2^) for this regression line was 0.85, which reflected the fact that the observed level of HSV-2 shedding in many guinea pigs did not conform perfectly to the quantity predicted by the equation y = 3.77–0.95x (Fig. 4C). However, the average level of vaginal HSV-2 shedding decreased in direct proportion to pan-HSV-2 IgG levels in all 4 immunization groups, within the standard error of the measurements (Fig. 4D; r^2^ = 0.98).

Regarding disease progression, naïve guinea pigs uniformly developed florid perivaginal disease and had to be sacrificed on or before Day 11 post-challenge (Fig. 4E). Guinea pigs immunized with gD-2 possessed low pan-HSV-2 IgG levels, and 3 of 4 developed florid perivaginal disease that required their sacrifice on or before Day 11 post-challenge (Fig. 4E). In contrast, guinea pigs immunized with the live HSV-2 viruses MS or 0ΔNLS possessed high pre-challenge pan-HSV-2 IgG levels, and 100% of these guinea pigs survived vaginal HSV-2 challenge without developing any visible symptoms of disease (Fig. 4E).

The results of vaginal HSV-2 challenge experiments in mice and guinea pigs was compared (Table 2). In both species, immunization with gD-2 elicited a significant increase in pan-HSV-2 IgG that was an average 10- to 20-fold above background, and which correlated with partial protection against vaginal HSV-2 challenge (Table 2). In contrast, mice or guinea pigs immunized with the live HSV-2 viruses MS or 0ΔNLS mounted pan-HSV-2 IgG antibody responses that were 30- to 40-fold greater than gD-2 immunized animals (Table 2). Likewise, mice or guinea pigs immunized with MS or 0ΔNLS shed an average 20- to 35-fold less HSV-2 per vagina relative to gD-2 immunized animals (Table 2). Collectively, these results indicated that increased pan-HSV-2 IgG levels in immunized mice and guinea pigs correlated with increased vaccine-induced protection against HSV-2 in terms of ***1.*** reduced vaginal shedding of the HSV-2 challenge virus and ***2.*** increased survival frequency.

**Table 2:** Pan-HSV-2 IgG antibody levels correlate with protection against vaginal HSV-2 MS challenge in mice and guinea pigs.

Immunogen a	Species^b^	log (pan- HSV-2 IgG)^c^	log decrease in vaginal HSV-2 shedding^d^	Survival frequency^e^
Medium (naïve)	mice	−0.1±0.1	0.0±0.1	0/10
	guinea pigs	0.1±0.1	0.0±0.2	0/5
GFP+alum/MPL	mice	−0.1±0.1	−0.4±0.3	0/10
	guinea pigs	ND^f^	ND	ND
gD-2+ alum/MPL	mice	1.0±0.1**	0.7±0.1*	1/10
	guinea pigs	1.3±0.2**	0.7±0.1*	1/4
HSV-2 MS (ACV-restrained)	mice	2.6±0.1**	1.9±0.2**	10/10††
	guinea pigs	2.4±0.1**	2.2±0.1**	5/5†
HSV-2 0ΔNLS	mice	2.8±0.1**	2.2±0.2**	10/10††
	guinea pigs	2.6±0.1**	2.3±0.3**	5/5†

a Animals were immunized with each immunogen, as described in Figures 3A and 4A.

b Naive and immunized mice correspond to animals presented in Figure 3; guinea pigs correspond to animals presented in Figure 4.

c Mean ± sem of log (pan-HSV-2 IgG) for mice correspond to x-variables in Figure 3C, and for guinea pigs correspond to x-variables in Figure 4C.

d Mean ± sem of log (reduction in vaginal HSV-2 shedding) in mice was derived from the y-variables presented in Figure 3C, and for guinea pigs was derived from the y-variables presented in Figure 4C.

e Frequency of animals that survived until Day 30 post-HSV-2 vaginal challenge.

f Not determined.

* p<0.05, as determined by one-way ANOVA and Tukey's post-hoc t-test comparing immunized versus naïve animals of the same species.

** p<0.001, as determined by one-way ANOVA and Tukey's post-hoc t-test comparing immunized versus naïve animals of the same species.

† p = 0.01, as determined by Fisher's Exact Test comparing the frequency of survival of immunized versus naïve animals of the same species.

†† p = 0.00001, as determined by Fisher's Exact Test comparing the frequency of survival of immunized versus naïve animals of the same species.

### HSV-2 Antiserum Alone Offers Weak Protection against HSV-2 MS Challenge

High levels of pan-HSV-2 IgG antibodies correlated with robust protection against HSV-2 MS challenge in mice immunized with several live HSV-2 vaccines. A final experiment was conducted to determine if adoptive transfer of HSV-2 antiserum recapitulated the level of protection against HSV-2 observed in mice immunized with the HSV-2 0ΔNLS virus.

To this end, strain 129 mice (n = 10) were immunized in their right and left rear footpads with 10^6^ pfu of HSV-2 0ΔNLS on Days 0 and 30, respectively. On Day 85, five immunized mice were sacrificed to collect HSV-2 antiserum, and naïve serum was harvested at this time from age-matched controls. On Day 90, naïve mice received an adoptive transfer of 0.25 ml pooled naïve serum or HSV-2 antiserum (n = 5 per group), and were then challenged with 100,000 pfu per eye of HSV-2 MS. Likewise, n = 5 mice immunized with HSV-2 0ΔNLS were also challenged with 100,000 pfu per eye of HSV-2 MS.

**Figure 5:**
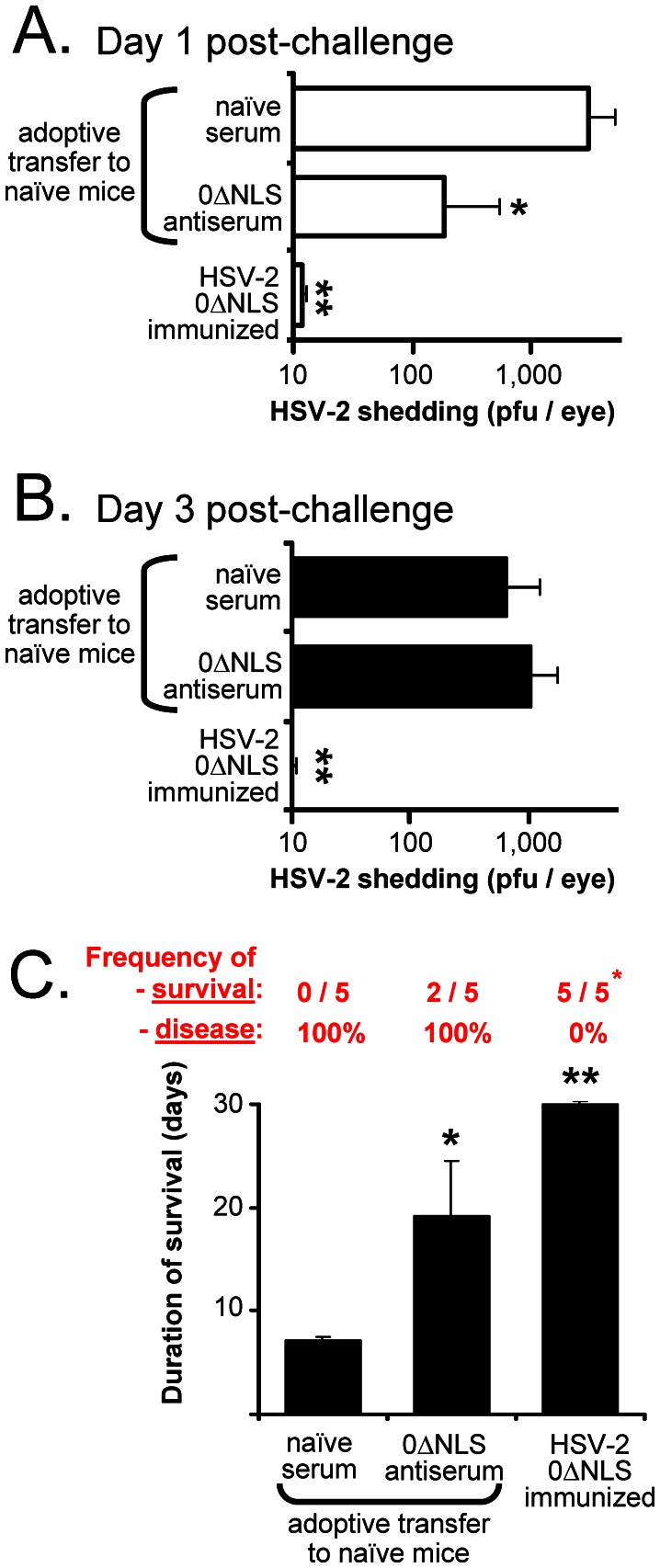
Adoptive transfer of HSV-2 antiserum provides limited protection against ocular HSV-2 MS challenge. Female, age-matched strain 129 mice received either ***i.*** an adoptive transfer of 0.25 ml naïve serum prior to challenge; ***ii.*** an adoptive transfer of 0.25 ml HSV-2 antiserum just prior to challenge; or ***iii.*** active immunization with the live HSV-2 0ΔNLS virus 90 and 60 days prior to challenge. Mice were challenged in both eyes with 100,000 pfu per eye of HSV-2 MS, and challenge virus shedding and disease onset were recorded. (**A and B**) Mean ± sem of HSV-2 shedding from mouse eyes on (**A**) Day 1 and (**B**) Day 3 post-challenge (n = 5 per group). (**C**) Mean ± sem duration of survival of each group of mice. Red numbers over each bar report the frequency of 'survival' and 'disease incidence' in each group of mice. Significant increases in the duration of survival relative to naïve mice are indicated by a single asterisk (*; p<0.05) or double asterisk (**; p<0.001), as determined by one-way ANOVA and Tukey's post-hoc t-test.

Ocular shedding of HSV-2 MS was compared. On Day 1 post-challenge, mice treated with naïve serum shed an average 3,000 per eye of HSV-2 MS, whereas mice treated with HSV-2 antiserum shed an average 16-fold less HSV-2 and this difference was significant (Fig. 5A). However, HSV-2 antiserum-treated mice and naïve serum-treated mice shed high and equivalent levels of HSV-2 on Day 3 post-ocular challenge (Fig. 5B). In contrast, mice immunized with HSV-2 0ΔNLS shed an average 300- and 60-fold less HSV-2 MS on Days 1 and 3, respectively, relative to naïve serum-treated mice (Fig. 5A, 5B).

Adoptive transfer of HSV-2 antiserum delayed, but did not prevent, the progression of HSV-2-induced pathogenesis. Specifically, 100% of naïve serum-treated mice succumbed to ocular HSV-2 challenge on Days 7 or 8 post-challenge (Fig. 5C). Two of 5 HSV-2 antiserum-treated mice survived ocular HSV-2 challenge, and as a group these mice survived for 19±5 days post-challenge (Fig. 5C). Although mice treated with HSV-2 antiserum survived significantly longer, these animals were not well protected. Specifically, 100% of HSV-2 antiserum-treated mice developed overt periocular fur loss and disease between Days 10 and 14 post-challenge, and 60% of these mice succumbed to challenge (Fig. 5C). In contrast, 100% of HSV-2 0ΔNLS-immunized mice survived without any overt signs of disease for 30 days post-challenge (Fig. 5C). Therefore, while pan-HSV-2 IgG antibody levels correlated with vaccine-induced protection against HSV-2 (Figs. 2–4), it is unlikely than anti-HSV-2 antibodies alone were the sole mediators of vaccine-induced protection against HSV-2 challenge.

## Discussion

The current study demonstrates that bloodstream levels of pan-HSV-2 IgG antibody in vaccinated mice and guinea pigs correlated with protection against HSV-2. We have not determined if other components of the adaptive immune response would also correlate with vaccine-induced protection against HSV-2. For example, HSV-2-specific T-cell frequency [51], [55], [56] or anti-HSV-2 IgA abundance in the vaginal mucosa [57] may provide better correlates of immunity for a HSV-2 vaccine. However, it should be noted that the utility of a *correlate of immunity* is not dependent on its role in mediating protection. Rather, a correlate of immunity is a screening tool whose utility lies solely in its ability to gauge the magnitude of vaccine-induced protection against a microbial pathogen. It remains to be determined if pan-HSV-2 IgG levels would be useful in gauging HSV-2 vaccine efficacy in human clinical trials.

### Relevance of Humoral Versus Cellular Immunity in Vaccine-induced Protection against HSV-2

The relevance of humoral versus cell-mediated immunity in vaccine-induced protection against HSV-2 remains incompletely defined. What is evident from decades of studies dating back to Oakes, 1975 [58] is that adoptively transferred anti-HSV antibodies or B-cells alone are not sufficient to prevent peripheral HSV-1 infection from progressing to fatal disease in immunodeficient nude or SCID mice [59], [60]; whereas, adoptively transferred T-cells are sufficient to allow immunodeficient animals to survive peripheral infection with low virulence strains of HSV-1 [59], [60]. Moreover, T-cells play a direct role in controlling HSV-1 and HSV-2 infections in sensory ganglia [44], [50], [61]–[65]. Thus, vaccine-induced protection against HSV-2 will almost certainly be dependent upon the T-cell response to HSV-2 antigens [66]–[70].

We would suggest that complete, vaccine-induced protection against HSV-2 genital herpes lesions will most likely be dependent upon a balanced B-cell (antibody) and T-cell response to HSV-2's antigens. Two lines of evidence support this hypothesis. First, SCID mice reconstituted with both B- and T-cells control HSV-1 infection significantly more rapidly than SCID mice reconstituted with T-cells alone (Fig. 1C in Ref. [60]); numerous investigators have reported similar findings with HSV-1 or HSV-2 [71]–[73]. Second, T-cells alone are slow to infiltrate sites of HSV-1 or HSV-2 challenge unless chemokines [47] or inflammatory stimuli [74] are used to artificially increase the rate of T-cell recruitment. In contrast, antibodies are ∼100 billion-fold smaller than T-cells, and may rapidly enter virus-infected tissues; hence, antibodies may act during the first 24 h to restrict HSV-2 replication and/or spread (Fig. 5A).

Against this background, we would propose that a logical function for anti-HSV-2 antibodies would be to serve as the first line of adaptive immune defense that triggers the pro-inflammatory events (e.g., complement cascade) that promote the rapid recruitment of T-cells into virus-infected tissues at the portal of HSV-2 entry (e.g., the vagina). While this hypothesis is consistent with the available evidence, we caution that it remains to be tested. Therefore, it will be of interest to explore this and other alternative hypotheses that may explain the respective roles of humoral and cellular immunity in vaccine-induced protection against HSV-2.

### Correlates of Immunity to HSV-2: Current Study Versus Earlier Findings

Previous attempts to identify correlates of immunity to HSV-2 have focused on immune responses to the immunogens under study; namely, gB and/or gD [1]–[7], [13], [75]. These approaches do not consider HSV-2's full complement of antigens. At least 20 viral proteins are known targets of the human B- and T-cell response to HSV-2 [49], [51], [52]. We would suggest that such glycoprotein-focused studies have not adequately considered that viral antigens other than gB-2 and gD-2 may also contribute to immunity to HSV-2.

Glycoprotein-centric correlates of immunity suggest that gB-2 and/or gD-2 subunit vaccines should be sufficient to prevent HSV-2 genital herpes in humans [2]–[5]. This prediction has not been borne out by the data from human clinical trials spanning the last 23 years [32]–[37]. It is our opinion that the pan-HSV-2 IgG metric is a more realistic correlate of immunity because it weighs the relative abundance of IgG antibodies against *all of HSV-2's antigens*, and thus is not contingent upon an assumption that the immune response to 1 or 2 specific proteins will necessarily provide an accurate gauge of immunity to HSV-2.

The results of the current study demonstrate that immunization with a gD-2 vaccine elicits a significant pan-HSV-2 IgG antibody response and a significant reduction in vaginal HSV-2 shedding (Table 2). However, animals immunized with *polyvalent* HSV-2 viruses mount an ∼30-fold greater pan-HSV-2 IgG response than gD-2-immunized animals, and likewise exhibit ∼25-fold lower vaginal HSV-2 shedding after challenge (Table 2). These results raise the possibility that, in addition to gD-2, immune responses directed against HSV-2's other 20 antigens may also contribute to the protective efficacy of a live HSV-2 vaccine. Continued studies will be required to further explore this hypothesis.

### Use of Regression Analysis to Detect a Correlate of Immunity to HSV-2

Several HSV-2 vaccine-challenge studies have attempted to measure protection against HSV-2 in terms of disease scores, survival, or weight gain after HSV-2 challenge [6], [76], [77]. We would suggest that non-parametric statistics (i.e., disease and survival) or tangential parameters (i.e., weight gain) are weak measures of the primary variable under study, *protection against HSV-2*. In contrast, reductions in HSV-2 challenge virus shedding are a precise measure of protection against HSV-2, and vary over an ∼500-fold range. The use of this robust measure of protection allowed linear regression analysis to be applied in the current study to determine if increased pan-HSV-2 IgG levels (x) correlated with protection against HSV-2 (y), as gauged by reductions in ocular or vaginal HSV-2 shedding (Fig. 2C, 3C, 4C).

Linear regression analysis is one of the most powerful statistical tools available to determine if a correlation exists between two variables. To the best of our knowledge, the current study is the first to apply regression analysis to detect a correlation between a parameter of the adaptive immune response and protection against HSV-2. This innovation was critical to the success of the current study. The ability to detect a correlation between two parameters by regression analysis is dependent on three variables: ***Variable 1.*** the number of matched x, y datum pairs in the data set; ***Variable 2.*** the precision of measurements of the x- and y-variables; and ***Variable 3.*** the range of Δx and Δy over which a correlation may be observed.

Regarding Variable 2, the flow cytometric assay introduced herein improved the precision and sensitivity of estimates of pan-HSV-2 IgG levels (Table 1, Fig. S2C), and thus improved the r^2^-value of the correlation relative to antibody-capture ELISA. This technical innovation enhanced our ability to detect a correlation between pan-HSV-2 IgG (x-variable) and reductions in HSV-2 challenge virus shedding (y-variable).

Regarding Variable 3, if we had focused exclusively on one vaccine modality such as the HSV-2 0ΔNLS vaccine, then the observed range of pan-HSV-2 IgG levels (Δx) would have been too narrow (∼5-fold) to detect a meaningful correlation (Fig. 3C, 4C). However, by employing six HSV-2 immunogens in three independent challenge experiments, we were able to expand the range of observed pan-HSV-2 IgG levels to an ∼500-fold range (Fig. 2C, 3C, 4C). Thus, the success of the current study was highly dependent on the use of a total n = 117 animals which collectively offered a 500-fold range of pan-HSV-2 IgG levels (Δx) over which we could test for proportional decreases in HSV-2 challenge virus shedding (y).

### Conclusion

The current study demonstrates that in vaccinated mice and guinea pigs, the pan-HSV-2 IgG antibody response to several vaccines varies in proportion to protection against HSV-2. It is possible that this same approach may provide a useful screening tool in human clinical trials of a HSV-2 vaccine. Based on the results, we would predict that a HSV-2 vaccine formulation that elicits the most potent and durable pan-HSV-2 IgG antibody response in humans should elicit the greatest protection against HSV-2 genital herpes. However, we acknowledge the caveat that the proposed utility of pan-HSV-2 IgG as a potential correlate of vaccine-induced protection against HSV-2 remains to be tested in humans. Therefore, it will be of interest to test this prediction in coming years, and determine if pan-HSV-2 IgG levels provide a useful correlate of vaccine-induced protection against HSV-2 in humans.

## Materials and Methods

### Ethics Statement

Mice and guinea pigs were handled in accordance with the National Institutes of Health Guide for the Care and Use of Laboratory Animals. This study was approved by the Southern Illinois University School of Medicine Laboratory Animal Care and Use Committee, and was performed as described under approved protocol 205-08-019.

### Cells and Viruses

Vero cells and U2OS cells were obtained from the American Type Culture Collection (Manassas, VA), and ICP0-complementing L7 cells were kindly provided by Neal Deluca (University of Pittsburgh; Ref. [78]). All cells were propagated in Dulbecco’s Modified Eagle’s medium (DMEM) supplemented with 5% fetal bovine serum (FBS), 100 U/ml penicillin G, and 100 mg/ml streptomycin, hereafter referred to as “complete DMEM.” Wild-type HSV-2 MS (ATCC) was propagated and titered on Vero cells. The HSV-2 *ICP0* ^−^ mutant viruses used in this study (HSV-2 0Δ810, 0Δ254, and 0ΔRING [53]) were propagated in U2OS cells and titered in ICP0-complementing L7 cells.

### HSV-2 Challenge Studies

A retrospective analysis of serum obtained two years earlier was performed in the current study (Fig. 2A and 3A). The details of these studies are described elsewhere [29], [53]. Prospective vaccine-challenge studies in guinea pigs are described in detail, as follows.

Female Hartley guinea pigs were obtained at an average weight of 250 g from Charles River (Wilmington, MA). On Day 0, guinea pigs were anesthetized by i.p. administration of xylazine (5 mg/kg) and ketamine (30 mg/kg), and were immunized via right, rear footpad injection of 100 µl containing ***1.*** complete DMEM (naïve), ***2.*** 2×10^6^ pfu HSV-2 0ΔNLS, ***3.*** 2×10^6^ pfu HSV-2 MS, or ***4.*** 5 µg recombinant glycoprotein D-2 (gD-2) antigen +20 µg monophosphoryl lipid A (Avanti Polar Biolipids)+Imject alum adjuvant (Thermo Scientific). The gD-2 antigen was expressed from a baculovirus vector [54] and has been used as a vaccine antigen in numerous studies [2], [7], [29]. The details of purification of this His-tagged gD-2 protein are described elsewhere [29]. Guinea pigs immunized with HSV-2 MS received 1 mg/ml oral acyclovir in their drinking water between Days 0 and 20 post-immunization to limit viral pathogenesis; 100% of guinea pigs survived their primary exposure to HSV-2 MS without developing overt signs of disease. Guinea pigs received an equivalent immunization in their left, rear footpads on Day 30 (per design shown in Fig. 4A). HSV-2 MS-immunized guinea pigs were not treated with acyclovir at the time of the second, booster immunization. Guinea pigs were bled on Day 75 post-immunization by saphenous vein puncture with a 25 g needle and blood was collected with a heparinized, Natelson blood collecting tube. The serum fraction was collected and stored at −80°C.

All guinea pigs were challenged with HSV-2 MS on Day 90, as follows. Prior to viral inoculation, guinea pigs were anesthetized by i.p. administration of xylazine (5 mg/kg) and ketamine (30 mg/kg). Naïve and immunized guinea pigs were vaginally challenged with wild-type HSV-2 MS by ***1.*** first clearing the mucus plug from the vagina with a cotton swab, ***2.*** twirling a second cotton swab inside the vaginal vault to further dry the walls of the vagina, and ***3.*** instilling the vaginal vault with 40 µl complete DMEM containing 2×10^6^ pfu of HSV-2 MS.

Viral titers in the vaginal vault of challenged guinea pigs were determined at 8 hours post-challenge (eclipse phase) and on Days 1, 2, 3, 4, 6, and 8 post-challenge by inserting and twirling a swab in the vaginal vault of guinea pigs, and transferring the tip into 0.4 ml complete DMEM. Viral titers were determined as described above. Guinea pigs were monitored daily, and animals that exhibited severe perivaginal ulceration were euthanized at the earliest possible time. The perivaginal region of all guinea pigs was photographed on Day 7 post-challenge. Surviving guinea pigs were euthanized on Day 30 post-challenge.

### Adoptive Transfer of HSV-2 Antiserum to Inbred Strain 129 Mice

Female strain 129 mice were obtained at 6- to 8-weeks of age from Charles River (Wilmingtion, MA). On Days 0 and 30, n = 10 mice were anesthetized by i.p. administration of xylazine (7 mg/kg) and ketamine (100 mg/kg), and were immunized via right and left rear footpad injection, respectively, of 50 µl containing 10^6^ pfu HSV-2 0ΔNLS. On Day 85, n = 5 HSV-2 0ΔNLS-immunized mice were sacrificed to harvest HSV-2 antiserum, and n = 5 age-matched, naïve mice were sacrificed to harvest naïve serum. On Day 90, naïve mice received an adoptive transfer of 0.25 ml pooled HSV-2 antiserum or 0.25 ml pooled naïve serum. Immediately following adoptive transfer, these n = 10 naïve mice were anaesthetized by i.p. administration of xylazine (7 mg/kg) and ketamine (100 mg/kg), and were challenged with 100,000 pfu per eye of HSV-2 MS. Likewise, n = 5 mice immunized with HSV-2 0ΔNLS (on Days 0 and 30) were anaesthetized and challenged at the same time with 100,000 pfu per eye of HSV-2 MS. HSV-2 MS shedding was monitored in these mice as described elsewhere [29].

### Antibody Capture ELISA to Enumerate Pan-HSV-2 IgG Antibody Levels in Serum

High-binding EIA 96-well plates (Costar, Corning, NY) were coated overnight at 4°C with 100 µl per well of sodium carbonate buffer (pH 9.6) containing 0.2 µg per ml total HSV-2 antigens. Total HSV-2 antigen was isolated from HSV-2 infected Vero cells, as follows: five 100-mm dishes of Vero cells (8 million cells per dish) were inoculated with 3 pfu per cell of HSV-2 MS and incubated at 37°C for 16 hours. Culture medium was aspirated from dishes, cells were rinsed with 5 ml PBS per dish, and cells were covered in 2 ml of sodium carbonate buffer (pH 9.6) per dish and frozen at −80°C. HSV-2 cell lysates were thawed and clarified by low-speed centrifugation to remove cell debris. The clarified supernatant had a protein concentration of 10 µg/ml, and was frozen in 0.2 ml aliquots. For each 96-well plate to be coated with HSV-2 antigen, a single aliquot of HSV-2 total antigen was diluted 1∶50 (0.2 µg per ml) and used to coat a high-binding EIA plate. After overnight coating with total HSV-2 antigen, wells were blocked for 2 hours with 400 µl of 2% dry milk dissolved in phosphate-buffered saline (PBS) +0.02% Tween-20 (polyoxyethylene-20-sorbitan monolaurate), hereafter referred to as PBS-T buffer. Each serum sample to be tested was diluted 2.5∶ 250 in PBS +1% fetal bovine serum +0.02% Tween-20. After discarding blocking buffer from ELISA plates, duplicate 100-µl samples of diluted serum were added to total HSV-2 antigen-coated wells and were incubated for 2 hours. ELISA plates were rinsed three times with an excess of PBS-T buffer prior to the addition of 100 µl secondary antibody diluted 1∶1500 in PBS-T buffer; the secondary antibody was alkaline phosphatase-conjugated goat anti-mouse IgG Fc fragment (Rockland Immunochemicals, Gilbertsville, PA). After allowing 1 hour, secondary antibody was rinsed from plates seven times with PBS-T buffer, and 200 µ1 of p-nitrophenyl phosphate substrate (Sigma Chemical Co., St. Louis, MO) was added to each well, and colorimetric development (OD_405_) was measured after a 30-minute incubation at room temperature. The quantitative relationship between abundance of log (pan-HSV-2 IgG) (x) and OD_405_ (y) was defined using a 0.33-log dilution series of HSV-2 antiserum and a hyperbolic tangent-based standard curve (Fig. S2A). The abundance of log (pan-HSV-2 IgG) in each serum sample was derived from OD_405_ values using a reciprocal hyperbolic arctangent equation of the form x = x_50_+ ΔX • arctan 
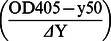
, as described elsewhere [53], [79].

### Flow Cytometry Assay to Enumerate Pan-HSV-2 IgG Levels in Mouse and Guinea Pig Serum

Single-cell suspensions of a mixture of HSV-2^+^ cells and uninfected (UI) cells were generated, as follows. Twelve 100-mm dishes were seeded with 7×10^6^ Vero cells per dish in complete DMEM, and six dishes were inoculated 6 hours later with 3 pfu per cell of HSV-2 MS. HSV-2^+^ Vero cells were harvested 12 hours after inoculation, and UI Vero cells were harvested in parallel at the same time. Both cell populations were dispersed by aspirating culture medium, rinsing each dish with 5 ml PBS, and adding 2 ml PBS +5 mM ethylene diamine tetraacetic acid (EDTA) pH 8.0. It should be noted that PBS +5 mM EDTA was sufficient to cause Vero cells to lift and detach from one another without the use of trypsin. In the case of HSV-2^+^ cells, the PBS +5 mM EDTA solution was supplemented with 1 µM carboxyfluorescein diacetate, succinimidyl ester (CFSE; Anaspec, Fremont, CA) to label HSV-2^+^ cells with a green fluorophore. Cells were incubated at room temperature on a rocking platform for 10 minutes until cells began to lift, and were then dispersed by trituration with the aid of a P-1000 pipettor. All dispersed UI cells were placed in a single 50-ml conical, and all dispersed HSV-2^+^ cells were placed in a second 50-ml conical, and both were centrifuged at 200×g for 5 minutes to pellet cells. Supernatants were decanted, cell pellets were resuspended in 12 ml PBS, and an equal volume of 2× fixative (7.4% formaldehyde +4% sucrose) was added. Cells were incubated in 1× fixative for 20 minutes, centrifuged, and resuspended in 24 ml of 90% methanol to permeabilize the cells. After a 10 minute incubation, cells were centrifuged, resuspended in PBS +3% fetal bovine serum (PBS-F), and cell clumps were removed by passage through a 40 µM, nylon mesh cell strainer (BD Biosciences, San Jose, CA) followed by passage through a 25-gauge needle. Cell density in single-cell suspensions of UI Vero cells and CFSE-labeled HSV-2^+^ cells was determined, and UI cells and HSV-2^+^ cells were combined in an approximate 2∶1 ratio. Cells were centrifuged, resuspended at a concentration of 1.25×10^6^ cells per ml in PBS-F-Ig block solution (i.e., PBS-F supplemented with 20 µg/ml each of donkey γ-globulin, goat γ-globulin, and human γ-globulin; Jackson Immunoresearch Laboratories, Inc., West Grove, PA). Aliquots of UI and HSV-2^+^ cells (400 µl; 500,000 cells) were placed in 1.7 ml microfuge tubes, and 2 µl of 1∶30 diluted serum was added to each cell suspenstion to achieve a net serum dilution of 1∶6,000. Cells were incubated at room temperature for 4 hours on a LabQuake rotisserie rotator to keep cells in suspension by rotation (Barnstead International, Dubuque, IA), and primary antibody was removed by two, sequential 1.25 ml PBS-F rinses, where a swinging bucket centrifuge was used to pellet cells and rinse supernatant was aspirated.

To enumerate the amount of IgG antibody bound to HSV-2^+^ versus UI cells, cells were incubated with a 1∶1,000 dilution of APC-conjugated goat-anti mouse IgG Fc fragment or APC-conjugated donkey anti-guinea pig IgG (H+L) (Jackson Immunoresearch Laboratories, Inc.). After a 1-hour incubation, excess secondary antibody was removed by three, sequential 1.25 ml PBS-F rinses. Cells were resuspended in a total volume of 0.2 ml PBS-F and analyzed by two-color flow cytometry in the FL1 and FL4 channels of an Accuri C6 flow cytometer using CFlow software (Accuri Cytometers, Inc., Ann Arbor, MI). On average, 125,000 events were recorded per sample; specifically, the flow cytometer was set to record events until 25,000 single HSV-2^+^ cells were included in the data set. Pan-HSV-2 IgG levels in each serum sample were calculated based on the difference in mean fluorescent intensity (ΔMFI) of 25,000 HSV-2^+^ cells versus ∼50,000 UI cells (Fig. 1). Background fluorescence was defined as the average ΔMFI-value observed in cell suspensions incubated with naïve serum.

### Mathematical and Statistical Analysis of Results

Unless otherwise specified, all values presented are the mean ± standard error of the mean (sem) of replicate samples. Viral titers were determined by microtiter plaque assay and were statistically analyzed on a logarithmic scale (e.g., log [pfu/vagina]). Infectious virus was not detectable in some ocular or vaginal swabs of well-immunized animals. In such events, the sample was assigned a value of 8 pfu per swab (i.e., the lower-limit of detection of the assay), such that all samples could be analyzed on a logarithmic scale. The significance of differences in multiple group comparisons was compared by one-way analysis of variance (ANOVA) followed by Tukey’s post hoc t-test using GraphPad Instat v3.10 software (GraphPad Software, Inc., La Jolla, CA). The significance of difference between two groups was performed using the “t-test assuming equal variances” function of Microsoft Excel. The significance of differences in survival frequency was determined by Fisher's Exact Test using freely available online software (Ref. [80]; http://quantpsy.org/fisher/fisher.htm).

All data were statistically analyzed using logarithmic values. Linear regression analysis was performed by the method-of-least-squares using the “regression” analysis function in Microsoft Excel, and was used to calculate the goodness-of-fit (r^2^-value) and the probability (p) that the y-variable did not change as a function of the x-variable. The coefficient-of-variance values reported in Table 1 were calculated for each HSV-2 antiserum dilution by the formula, 100× (standard deviation of triplicate samples ÷ mean of triplicate samples). The reported values in Table 1 represent the mean ± sem coefficient-of-variance for all HSV-2 antiserum dilutions in the linear range of the assay (i.e., 1∶21–1∶1,000 dilutions for the neutralization assay; 1∶100–1∶100,000 dilutions for the antibody capture ELISA; and 1∶6,000–1∶6,000,000 dilutions for the flow cytometry assay).

## Supporting Information

Figure S1 **Comparison of three methods to measure anti-HSV-2 antibody levels.** **(A)** HSV-2 neutralizing activity in a 0.33-log dilution series of mouse HSV-2 antiserum. Neutralizing antibody titer is reported as the mean ± sem of n = 3 replicates per dilution. **(B)** Antibody capture ELISA-based measurement of pan-HSV-2 IgG antibody levels in a 0.33-log dilution series of HSV-2 antiserum (mean ± sem of n = 3 replicates per dilution). **(C)** Flow cytometry-based measurement of pan-HSV-2 IgG antibody levels in a 0.33-log dilution series of HSV-2 antiserum (mean ± sem of n = 3 replicates per dilution). The dashed lines represents the lower limit of detection of each assay.
(TIF)

Figure S2 **Antibody-capture ELISA versus flow cytometry measurement of pan-HSV-2 IgG levels in mouse serum.** **(A)** Standard curve of antibody-capture ELISA. Open circles indicate the colorimetric development (OD_405_) observed in ELISA wells that received 0.33-log dilutions of HSV-2 antiserum (mean ± sd; n = 4 per dilution). The sigmoidal relationship between OD_405_ and log (pan-HSV-2 IgG) was precisely described using the hyperbolic tangent equation shown (r^2^ = 1.00), and a reciprocal hyperbolic arctangent equation (defined in Methods) was used to derive pan-HSV-2 IgG levels in test serum samples from the OD_405_ values observed in ELISA. **(B)** For each mouse (one symbol per mouse), the average amount of infectious HSV-2 shed on Days 1, 2, and 3-post ocular challenge (y-axis) was plotted as a function of the pre-challenge pan-HSV-2 IgG levels, as estimated by ELISA (x-axis). The solid black line represents the best-fit linear regression model, y = 3.05–0.57x, for the 48 matched datum pairs. (**C**) ELISA- versus flow cytometry-estimates of log (pan-HSV-2 IgG) are plotted as x,y-datum pairs relative to a 0−log “line of equivalence.” Datum points beyond the “+1 log” reference line indicate serum samples in which flow cytometry estimates of pan-HSV-2 IgG levels were 1 logarithm greater than the ELISA estimate of pan-HSV-2 IgG for the same serum sample.
(TIF)
