# Supplementary figures and images for: Pan-HSV-2 IgG Antibody in Vaccinated Mice and Guinea Pigs Correlates with Protection against Herpes Simplex Virus 2
Source: PLoS One. 2013 Jun 6;8(6):e65523. doi: 10.1371/journal.pone.0065523 (PMC3675040; doi:10.1371/journal.pone.0065523)

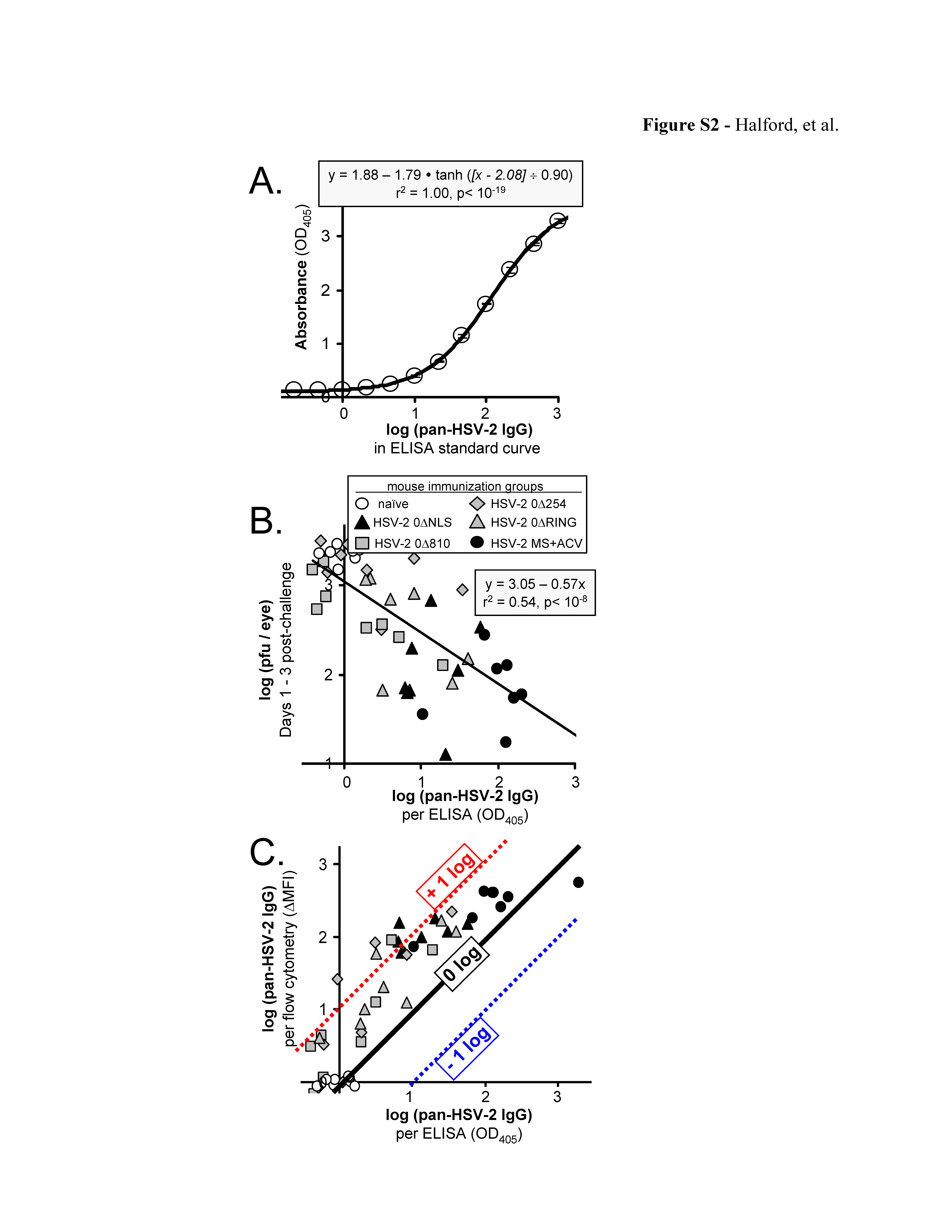

Supplement: Figure S2 — Antibody-capture ELISA versus flow cytometry measurement of pan-HSV-2 IgG levels in mouse serum. (A) Standard curve of antibody-capture ELISA. Open circles indicate the colorimetric development (OD405) observed in ELISA wells that received 0.33-log dilutions of HSV-2 antiserum (mean ± sd; n = 4 per dilution). The sigmoidal relationship between OD405 and log (pan-HSV-2 IgG) was precisely described using the hyperbolic tangent equation shown (r2 = 1.00), and a reciprocal hyperbolic arctangent equation (defined in Methods) was used to derive pan-HSV-2 IgG levels in test serum samples from the OD405 values observed in ELISA. (B) For each mouse (one symbol per mouse), the average amount of infectious HSV-2 shed on Days 1, 2, and 3-post ocular challenge (y-axis) was plotted as a function of the pre-challenge pan-HSV-2 IgG levels, as estimated by ELISA (x-axis). The solid black line represents the best-fit linear regression model, y = 3.05–0.57x, for the 48 matched datum pairs. (C) ELISA- versus flow cytometry-estimates of log (pan-HSV-2 IgG) are plotted as x,y-datum pairs relative to a 0−log “line of equivalence.” Datum points beyond the “+1 log” reference line indicate serum samples in which flow cytometry estimates of pan-HSV-2 IgG levels were 1 logarithm greater than the ELISA estimate of pan-HSV-2 IgG for the same serum sample. (TIF) [file pone.0065523.s002.tif]
